# Response of Korean pine’s functional traits to geography and climate

**DOI:** 10.1371/journal.pone.0184051

**Published:** 2017-09-08

**Authors:** Yichen Dong, Yanhong Liu

**Affiliations:** Key Laboratory for Forest Resources and Ecosystem Processes of Beijing, Beijing Forestry University, Beijing, China; Chinese Academy of Forestry, CHINA

## Abstract

This study analyzed the characteristics of Korean pine (*Pinus koraiensis*) functional trait responses to geographic and climatic factors in the eastern region of Northeast China (41°–48°N) and the linear relationships among Korean pine functional traits, to explore this species’ adaptability and ecological regulation strategies under different environmental conditions. Korean pine samples were collected from eight sites located at different latitudes, and the following factors were determined for each site: geographic factors—latitude, longitude, and altitude; temperature factors—mean annual temperature (MAT), growth season mean temperature (GST), and mean temperature of the coldest month (MTCM); and moisture factors—annual precipitation (AP), growth season precipitation (GSP), and potential evapotranspiration (PET). The Korean pine functional traits examined were specific leaf area (SLA), leaf thickness (LT), leaf dry matter content (LDMC), specific root length (SRL), leaf nitrogen content (LNC), leaf phosphorus content (LPC), root nitrogen content (RNC), and root phosphorus content (RPC). The results showed that Korean pine functional traits were significantly correlated to latitude, altitude, GST, MTCM, AP, GSP, and PET. Among the Korean pine functional traits, SLA showed significant linear relationships with LT, LDMC, LNC, LPC, and RPC, and LT showed significant linear relationships with LDMC, SRL, LNC, LPC, RNC, and RPC; the linear relationships between LNC, LPC, RNC, and RPC were also significant. In conclusion, Korean pine functional trait responses to latitude resulted in its adaptation to geographic and climatic factors. The main limiting factors were precipitation and evapotranspiration, followed by altitude, latitude, GST, and MTCM. The impacts of longitude and MAT were not obvious. Changes in precipitation and temperature were most responsible for the close correlation among Korean pine functional traits, reflecting its adaption to habitat variation.

## Introduction

By revealing plants’ adaptation to changes in environmental conditions during their growth, functional traits provide a clear and convenient quantitative evaluation method [1], whose advantages are attractive for ecological studies at various scales. For example, some studies have focused on functional traits’ diversity and species richness at the global and regional scales [2,3], others have compared the effects of various environmental conditions on plant traits [4,5,6], and yet others have used functional traits to evaluate regional forest community structure or ecosystem dynamics [7,8]. In recent years, the plant functional trait theory continued to improve with the establishment of the worldwide leaf-economics spectrum [9], global database of plant traits [10], and use of these data and methods to develop new areas of ecological research such as the plant-economics spectrum [11,12,13]. At the same time, additional research focused on the rationale behind the mechanisms of plant functional traits [14,15], as well as on the internal driving forces such as plant adaptability, ecological strategy, and response regulation [16,17,18]. The results of these theoretical studies led to the development and improvement of studies considering plants’ functional traits [19,20,21].

In the above theoretical studies, there is a key question that needs to be answered: How do plant functional traits quantitatively explain the adaptation of habitat variability, caused by changes in geographic and climatic conditions, in individual plants, populations, and communities [22]? One possible way is through exploring the characteristics of various plant functional trait responses to different habitats to verify the effects of habitat heterogeneity on populations and communities [23,24,25], as well as on biodiversity and species richness [26,27]. The effects of habitat heterogeneity caused by changes in latitude on species distribution and diversity have continuously received attention [28,29,30]. Some studies have shown that from low to high latitudes, changes in climatic factors such as temperature, precipitation, and evapotranspiration led to changes in ecosystem productivity and decreased biodiversity and species richness [27]. On the other hand, because of limitations caused by geomorphological factors such as mountains, deserts, and islands, the effects of changes in latitude appear to be diverse and complex, and their mechanisms are divergent and controversial [31,32]; therefore, the regularity and reliability of these effects need to be verified.

Korean pine (*Pinus koraiensis*) is an important timber species, with edible and ornamental functions, is a natural key protected wild plant in China, and is listed as an endangered species in 2013 by IUCN. Being the dominant tree species in this area, Korean pine plays a key role in forest succession, forest community structure dynamics, and biodiversity in Northeast China [33,34,35,36]. Previous studies of Korean pine have focused on the diversity of community in the process of succession and the response of community traits of habitat heterogeneity. Studies on the relationship between individual traits and environmental climatic factors are rare, and the use of functional traits to address issues such as plants’ environmental adaption mechanisms and the resource allocation strategy they adopt along latitude gradients require further research. It is of great significance to carry out such research for the renewal, distribution, and effective protection of Korean pine. Such studies can provide quantitative explanations for the response strategy of plants to the latitude gradient and the mechanism of adaption to a specific habitat resulting from particular geomorphological factors [37].

This study analyzed the functional traits of Korean pine at eight latitudes in northeastern China, and mainly answered the following question: is variability of the latitude heterogeneity of the functional traits of Korean pine the same? Further, we explored the relationship of Korean pine with latitude gradient and geographical and climatic factors. Thus, we aimed to determine whether individual trait responses to latitude variation in Korean pine were identical or similar to community responses to latitude changes and significant for explaining the relationships between individuals and the community [38]. The characteristics of community trait responses of the mixed coniferous broad-leaved forest dominated by Korean pine are crucial for evaluating the functions of the forest ecosystem, biodiversity, and species richness in this region [39,40,41]. Nevertheless, studies on Korean pine functional trait responses to latitude gradients and geographic and climatic factors are rare. Therefore, by exploring the regulatory mechanism of Korean pine traits, we not only verified the applicability of plant functional trait theory but also provided an experimental basis for the study of the community structure and dynamics of Korean pine-dominated mixed coniferous broad-leaved forests.

## Materials and methods

### Study sites

**Fig 1:**
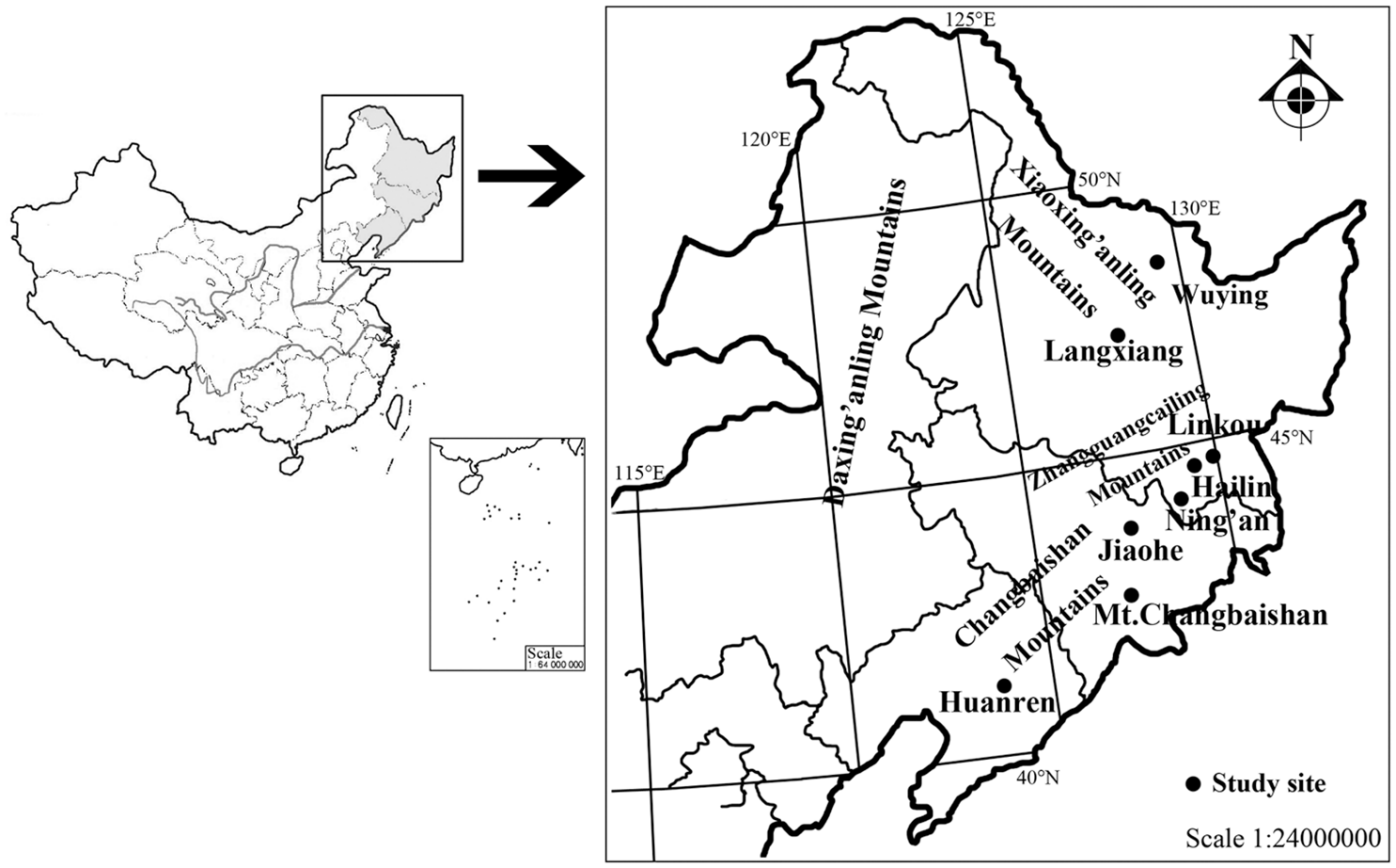
A geography of sampling sites in Northeast China. The sites include: Huiren (HR,41°20′N); Changbaishan (CBS, 42°23′N); Jiaohe (JH,43°58′N); Ning’an (N’A, 44°11′N); Hailin (HL, 44°33′N); Linkou (LK, 45°05′N); Langxiang (LX, 46°41′N); and Wuying (WY, 48°07′N). Map from National administration of surveying, mapping and geoinformation.

An overview of the geography of each sampling site is shown in Fig 1. Korean pine is mainly distributed in the eastern mountains of Northeast China (including Heilongjiang, Jilin, and Liaoning provinces), although it ranges between 34°N and 52°N and between 124°E and 135°E, along the eastern edge of the Eurasian continent. The northwestern side of this species distribution borders the Mongolian and the Siberian plateaus and its southeastern side is adjacent to the Japan, Bohai, and Yellow seas [42,43]. In this region, which is affected by the East Asian high-altitude monsoon climate and maritime air masses, the winter is cold and dry and the summer is hot and rainy. Because of geographic and climatic influences, in Northeast China, forest types change along a south–north gradient from warm-temperate deciduous broadleaf forest to temperate broadleaf and mixed forest, and to cold-temperate coniferous forest. From east to west, the area gradually changes from humid area to semi-humid and to semi-arid. The vegetation types are clearly distinct among forests and grasslands [44].

The eight sites where survey and sampling were conducted for the present study covered the main distribution areas of natural Korean pine (41°16′N–48°07′N; 124°49′N–130°02′E), being representative of its growth and development in Northeast China. These sites comprised nature reserves and forest areas with well-preserved Korean pine community structure and resources and with limited human interference, and an overview of the geography and climate of each sampling site is shown in Table 1. These regions preserved a relatively complete natural Korean pine community, suitable for carrying out studies on the effects of the geographic and climatic factors on Korean pine functional traits.

**Table 1:** The main geographic and climatic factors of the sampling sites.

Site	Latitude (°N)	Longitude (°E)	Altitude (m)	MAT (°C)	GST (°C)	MTCM (°C)	AP (mm)	GSP (mm)	PET (mm)	Region	Vegetation type
Huanren	41°21′	124°55′	633–703	3.7	13.2	-15.8	893.1	814.5	555.3	Laotudingzi National Nature Protection Zone, Liaoning	Changbaishan flora, broad-leaved mixed forest
Changbaishan	42°23′	128°05′	787.7	2.1	11.7	-17.5	814.3	740.4	520.3	Changbaishan National Nature Protection Zone, Jilin	Changbaishan flora, broad-leaved mixed forest
Jiaohe	43°58′	127°43′	390–450	2.8	13.2	-18.3	697.4	640.5	561.5	The advanced forest farm of the Forestry Experimental Zone Administration Bureau, Jilin	Changbaishan flora, secondary conifer and broad-leaved mixed forest
Ning'an	44°11′	128°34′	413–961	-2.9	9.8–13.1	-20.3	754.4	689.7	492	Xiaobeihu Korean Pine Protection Zone, Heilongjiang	Changbai larch forest, broad-leaved mixed forest
Hailin	44°33′	128°43′	354–820	0.3–2.8	12.1	-19.6	681.4	625.4	533.4	Heilongjiang Dahailin Forestry Bureau, Heilongjiang	Zhanggunagcai flora, broad-leaved mixed forest
Linkou	45°05′	130°02′	415.9	1.8	12.5	-19.6	607.4	558.3	547.5	Heilongjiang Linkou Forest Bureau, Heilongjiang	Zhanggunagcai flora, broad-leaved mixed forest
Langxiang	46°41′	129°04′	390.9	0.8	12	-21.9	566.6	527	540.2	The teaching and experimental base of Beijing Forestry University, Heilongjiang	Xiaoxing'anling flora, broad-leaved mixed forest
Wuying	48°07′	129°14′	330.5	-0.1	11.7	-23.6	500.7	470.8	536.3	Fenglin National Nature Protection Zone,Heilongjiang	Xiaoxing'anling flora, broad-leaved mixed forest

MAT, mean annual temperature; GST, growing season mean temperature; MTCM, mean temperature of the coldest month; AP, annual precipitation; GSP, growing season precipitation; PET, potential evapotranspiration.

### Ethics statement

This study was approved by the Resource Protection Office of Laotudingzi National Nature Protection Zone, Huanren, Forestry Department of Liaoning Province; Changbaishan National Nature Protection Zone, Changbaishan, Jilin Province and Jilin Changbai Mountain Forest Ecosystem National Field Scientific Observation Station; the advanced forest farm of the Forestry Experimental Zone Administration Bureau, Jiaohe, Forestry Department of Jilin Province; Resource Protection Office of Xiaobeihu Korean Pine Protection Zone, Ning’an, Forestry Department of Heilongjiang Province; Resource Protection Office Hailin Forestry Bureau, Mudanjiang, Forestry Department of Heilongjiang Province; Resource Protection Office Linkou Forest Bureau, Mudanjiang, Department of Heilongjiang Province; the teaching and experimental base of Beijing Forestry University, Langxiang, Heilongjiang Province; and Fenglin National Nature Protection Zone, Wuying, Forestry Department of Heilongjiang Province.

### Data

#### Climatic data collection

Sampling sites were located in remote mountain forests in Northeast China lacking weather stations to collect accurate information. Therefore, regional climatic factors at different latitudes were calculated using a climate equation (see [40] for details), which was based on longitude, latitude, and altitude data to calculate temperature or precipitation:

T(or P)=a+b Latitude +c Longitude+d Altitude (1)

where T (or *P*) is the average monthly temperature (or precipitation), and *a*, *b*, *c*, and *d* are constants. This equation was developed based on the summarized relationship between geoclimatic information and forest vegetation types in China [40,45], and its applicability and reliability for estimating climate and precipitation in Northeast China has long been verified [31,40,46,47,48]. In the present study, longitude, latitude, and altitude data from the eight sampling sites were applied to the equation to obtain climate data. For analytical convenience, climatic factors were divided into three groups (Table 1): geographic factors, namely latitude, longitude, and altitude; temperature factors: mean annual temperature (MAT), growing season mean temperature (GST), and mean temperature of the coldest month (MTCM); and moisture factors: annual precipitation (AP), growing season precipitation (GSP), and potential evapotranspiration (PET).

#### Trait measurement

Sampling was conducted from June to August 2015. A global positioning system (GPS) (UniStrong G120BD handheld GBS, Beijing, China) was used to collect longitude, latitude, and altitude information from each sampling site, and three replicates were taken from each sampling site. Ten to 20 plants were selected at each replication site, and their current-year leaves and underground fine roots were collected (for details of the functional traits measured and the methods used, see Table 2).

**Table 2:** Measuring methods of the functional traits of Korean pine.

Functional trait	Abbr	Unit	Measurement methods
Specific Leaf Area	SLA	mm^2^· mg^-1^	$\text{SLA}=\frac{\text{Leaf area}}{\text{Leaf dry weight}}$
Leaf thickness	LT	mm	A vernier caliper was used to measure leaf thickness
Leaf dry matter content	LDMC	mg·g^-1^	$\text{LDMC}=\frac{\text{Leaf dry weight}}{\text{Leaf fresh weight}}$
Specific root length	SRL	mm·mg^-1^	$\text{SRL}=\frac{\text{Root length}}{\text{Root dry weight}}$
Leaf nitrogen content	LNC	mg·g^-1^	The Kjeldahl method was adopted for measuring these contents
Leaf phosphorus content	LPC	mg·g^-1^	the molybdenum/antimony photometric method was adopted for measuring these contents
Root nitrogen content	RNC	mg·g^-1^	The Kjeldahl method was adopted for measuring these contents
Root phosphorus content	RPC	mg·g^-1^	the molybdenum/antimony photometric method was adopted for measuring these contents

Some steps were followed before calculating the traits. (1) SLA, the pine leaves were divided into 5 equal parts (cylinders); the external surface (photoradiation surface) of each cylinder was defined as the leaf area. Leaves were scanned and leaf area was calculated using Image-Pro Plus 6.0 (Media Cybernetics, Bethesda, MD, USA). Leaves were then oven-dried at 65°C for 72 h. (2) SRL, seedlings were cut at the transition between the stem and the root (where the tree bark texture changed). The roots were set aside for 24 h, dirt was washed off, and were then placed at room temperature for air-drying before scanning. Overlapping parts were split to facilitate scanning, and root length (sum of the taproot and lateral roots) was calculated using Image-Pro Plus 6.0. Roots were oven-dried at 65°C for 72 h and weighed to obtain root dry weight.

#### Data analysis

Data regarding Korean pine functional traits from the eight sampling sites were analyzed by SPSS 18.0 for Windows, using one-way analysis of variance (ANOVA) to determine whether differences among the mean values of traits at each sampling site were significant (*P* < 0.01). Values with significant differences were further examined by Duncan’s consistency subset. Linear regression was performed using Origin pro 2016, with the least squares method, and all data for trait analysis were log_10_-transformed. Figures were prepared in SPSS 18.0 for Windows, Origin pro 2016, and Microsoft Excel 2010.

## Results

### Korean pine functional trait responses to latitude

**Fig 2:**
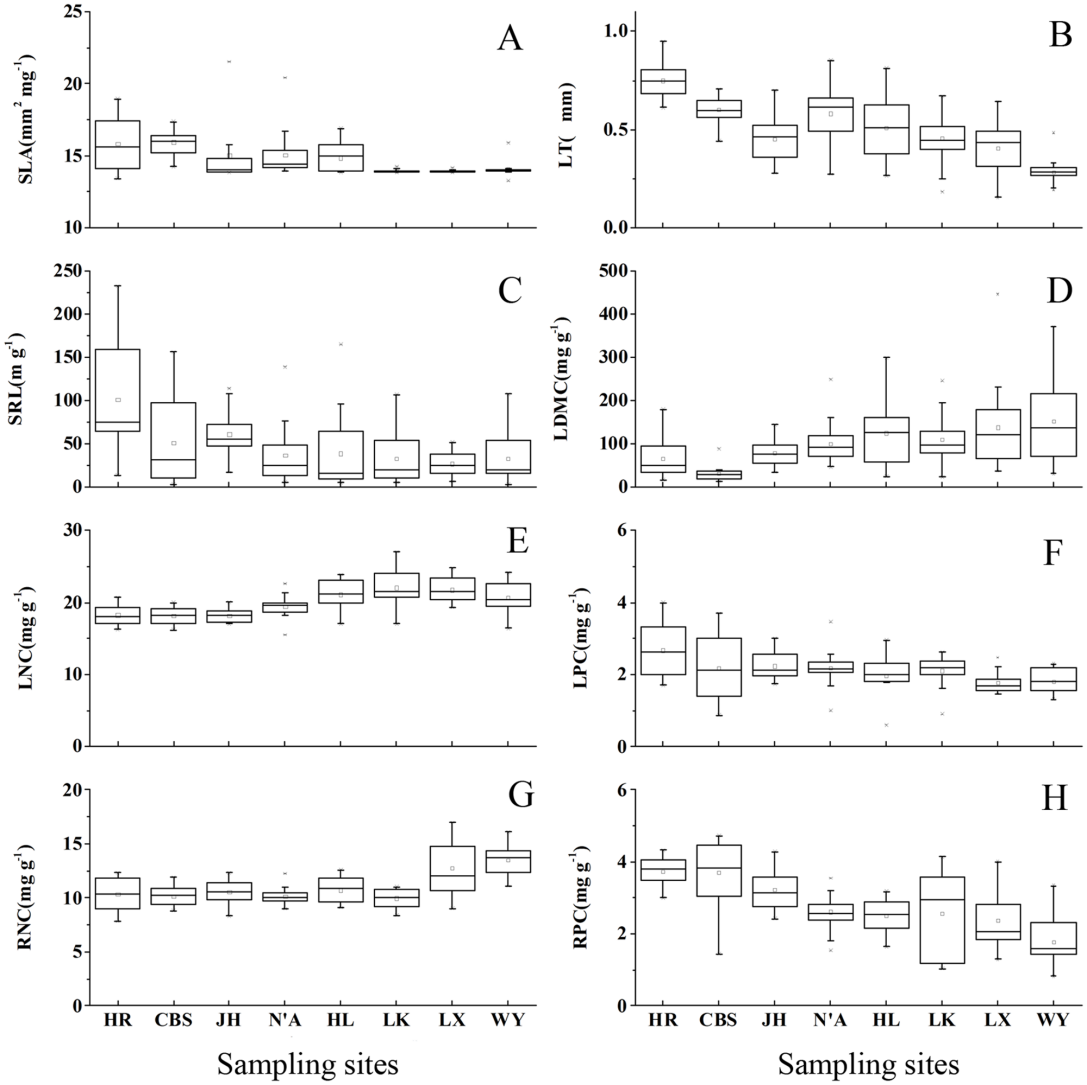
Trends in Korean pine’s functional traits from eight sampling sites at different latitudes. Data were analyzed using One-Way ANOVA to determine if the mean value of each trait differed among sampling sites at *P* < 0.01. In each chart (A~H), the Y-axis indicates the functional trait and the X axis indicates the sampling site. Functional traits: SLA, specific leaf area; LT, leaf thickness; LDMC, leaf dry matter content; SRL, specific root length; LNC, leaf nitrogen content; LPC, leaf phosphorus content; RNC, root nitrogen content; and RPC, root phosphorus content. Sampling sites: HR, Huiren (41°20′N); CBS, Changbaishan (42°23′N); JH, Jiaohe (43°58′N); N’A, Ning’an (44°11′N); HL, Hailin (44°33′N); LK, Linkou (45°05′N); LX, Langxiang (46°41′N); and WY, Wuying (48°07′N).

Changes in Korean pine functional trait responses to latitude are shown in Fig 2. Mean SLA varied from 13.95–15.97 mm^2^ mg^-1^, with the lowest SLA value (13.28 mm^2^ mg^-1^) appearing in Wuying (WY, 48°07′N) and the highest (21.53 mm^2^ mg^-1^) in Jiaohe (JH, 43°23′N). Thus, SLA values decreased with increasing latitude. The range of mean LT was 0.29–0.75 mm, and its lowest value (0.15 mm) was observed in Langxiang (LX, 46°41′N), whereas the highest (0.95 mm) was observed in Huiren (HR, 41°20′N). Thus, LT values also decreased with increasing latitude. Mean LDMC varied from 31.37 to 152.90 mg g^-1^; the lowest LDMC value (13.98 mg g^-1^) was observed in Changbaishan (CBS, 42°23′N) and the highest (392.98 mg g^-1^) in Langxiang (LX, 46°41′N). Thus, values increased with increasing latitude. Mean SRL ranged 21.12 to 101.87 m g^-1^. The lowest SRL value (3.17 m g^-1^) appeared in Changbaishan (CBS, 42°23′N) and the highest (232.55 m g^-1^) in Huiren (HR, 41°20′N), revealing that SRL decreased as latitude increased. Mean LNC ranged between 18.15 and 22.07 mg g^-1^, the lowest LNC value (15.51 mg g^-1^) was observed in Ning’an (N’A, 44°11′N), and the highest (27.02 mg g^-1^) in Linkou (LK, 45°05′N). Thus, values increased with increasing latitude. Mean LPC varied from 1.78 to 2.68 mg g^-1^; the lowest LPC (0.6 mg g^-1^) was observed in Hailin (HL, 44°33′N) and the highest (4.00 mg g^-1^) in Huiren (HR, 41°20′N), indicating that values slightly decreased with increasing latitude. Mean RNC values varied within 9.98 to 13.50 mg g^-1^, with the lowest RNC (7.82 mg g^-1^) appearing in Huiren (HR, 41°20′N) and the highest (17.00 mg g^-1^) in Langxiang (LX, 46°41′N); RNC values increased with increasing latitude. The range of mean RPC was 1.79–3.75 mg g^-1^. The lowest RPC (0.82 mg g^-1^) was observed in Wuying (WY, 48°07′N) and the highest (4.72 mg g^-1^) in Changbaishan (CBS, 42°23′N). Thus, RPC values decreased as latitude increased. Differences among Korean pine functional traits at the eight sampling sites were significant (*P* < 0.01), suggesting that latitude variation affected Korean pine.

### Relationships of functional traits of Korean pine with geographic and climatic factors

The results of the linear correlation analysis between Korean pine and geographic and climatic factors are shown in Table 3. The number of Korean pine samples collected from the eight sampling sites varied between 139 and 240. The values of LT, LNC, LPC, RNC, RPC, and SRL were significantly correlated to latitude (*P* < 0.05), and LT, LPC, RPC, and SRL showed a negative linear relationship with latitude (*R*^*2*^ ranged between 3% and 13%). Longitude showed a negative linear correlation with LDMC and SRL (*P* < 0.05), but did not show a linear relationship with other traits and *R*^*2*^ was less than 3%. Altitude showed significant correlations with all traits (*P* < 0.05), and presented a negative linear relationship with LDMC (*R*^*2*^ ranging between 3% and 21%). Although MAT showed a negative linear relationship with RNC, it had a positive linear relationship with RPC (*P* < 0.05) and an *R*^*2*^ around 4%. Significant linear relationships were found between GST and SLA, LT, LDMC, LNC, LPC, and SRL (*P* < 0.05), with *R*^*2*^ ranging between 2% and 10%. Although MTCM was positively correlated with LT, LNC, LPC, RNC, and RPC (*P* < 0.05), it was negatively correlated with LNC and RNC, with *R*^*2*^ ranging between 2% and 11%. Except for SLA, all traits presented positive linear correlations with AP and GSP (*P* < 0.05), with *R*^*2*^ ranging from 2–12% and 2–11%, respectively. Except for RNC, significant correlations were found between PET and all other traits (*P* < 0.05), and PET showed negative linear relationships with LT, LPC, RPC, and SRL, with *R*^*2*^ ranging between 2% and 13%. The above analyses indicated that latitude, altitude, GST, MTCM, AP, GSP, and PET had obvious effects on Korean pinetraits.

**Table 3:** Linear correlations between Korean pine functional traits and geographic and climatic factors.

*X*	*Y*	n	*R*^*2*^	*P*	*Slope*	*Intercept*
Latitude	logSLA	238	0.015	0.059	-0.002(-0.005, 0.000)	1.269(1.166,1.372)
	logLT	238	0.104	0.000	-0.025(-0.035,-0.016)	0.804(0.382,1.226)
	logLDMC	238	0.013	0.072	0.018(-0.002,0.037)	1.098(0.229,1.966)
	logLNC	139	0.083	0.000	0.007(0.004,0.010)	0.989(0.856,1.121)
	logLPC	139	0.038	0.003	-0.017(-0.029,-0.006)	1.005(0.502,1.507)
	logRNC	139	0.115	0.000	0.013(0.008,0.018)	0.464(0.257,0.671)
	logRPC	139	0.133	0.000	-0.030(-0.039,-0.020)	1.737(1.307,2.167)
	logSRL	178	0.030	0.007	-0.035(-0.061,-0.010)	3.119(1.984,4.255)
Longitude	logSLA	238	0.007	0.194	0.002(-0.001,0.005)	0.891(0.470,1.313)
	logLT	238	0.002	0.450	0.005(-0.009,0.020)	-1.021(-2.838,0.795)
	logLDMC	238	0.018	0.040	-0.029(-0.056,-0.001)	5.593(2.060,9.126)
	logLNC	139	0.010	0.123	0.003(-0.001,0.008)	0.860(0.298,1.422)
	logLPC	139	0.001	0.584	-0.005(-0.021,0.012)	0.808(-1.281,2.896)
	logRNC	139	0.009	0.154	0.005(-0.002,0.012)	0.399(-0.495,1.292)
	logRPC	139	0.010	0.116	-0.012(-0.026,0.003)	1.919(0.046,3.793)
	logSRL	178	0.029	0.008	-0.049(-0.085,-0.013)	7.831(3.199,12.463)
Altitude	logSLA	238	0.055	0.000	0.000(0.000,0.000)	1.144(1.130,1.159)
	logLT	238	0.209	0.000	0.000(0.000,0.000)	-0.539(-0.596,-0.482)
	logLDMC	238	0.107	0.000	-0.001(-0.001,0.000)	2.194(2.063,2.325)
	logLNC	139	0.139	0.000	0.000(0.000,0.000)	1.356(1.338,1.374)
	logLPC	139	0.109	0.000	0.000(0.000,0.000)	0.048(-0.021,0.117)
	logRNC	139	0.099	0.000	0.000(0.000,0.000)	1.121(1.091,1.151)
	logRPC	139	0.121	0.000	0.000(0.000,0.000)	0.250(0.189,0.312)
	logSRL	178	0.101	0.000	0.001(0.000,0.001)	1.158(1.002,1.314)
MAT	logSLA	238	0.001	0.575	-0.001(-0.005,0.003)	1.172(1.164,1.179)
	logLT	238	0.002	0.465	0.006(-0.010,0.023)	-0.333(-0.367,-0.299)
	logLDMC	238	0.005	0.281	-0.018(-0.050,0.015)	1.921(1.855,1.987)
	logLNC	139	0.006	0.218	-0.003(-0.008,0.002)	1.307(1.296,1.317)
	logLPC	139	0.000	0.825	-0.002(-0.021,0.017)	0.230(0.192,0.269)
	logRNC	139	0.039	0.002	-0.013(-0.021,-0.005)	1.068(1.052,1.084)
	logRPC	139	0.041	0.002	0.027(0.011,0.044)	0.376(0.342,0.410)
	logSRL	178	0.001	0.728	-0.008(-0.051,0.035)	1.558(1.471,1.645)
GST	logSLA	238	0.035	0.004	-0.008(-0.014,-0.003)	1.269(1.202,1.335)
	logLT	238	0.066	0.000	-0.048(-0.071,-0.025)	0.260(-0.021,0.542)
	logLDMC	238	0.099	0.000	0.122(0.073,0.171)	0.392(-0.201,0.985)
	logLNC	139	0.025	0.014	0.009(0.002,0.017)	1.190(1.101,1.279)
	logLPC	139	0.046	0.001	-0.046(-0.073,-0.019)	0.786(0.459,1.113)
	logRNC	139	0.001	0.655	0.003(-0.009,0.015)	1.015(0.872,1.159)
	logRPC	139	0.002	0.503	-0.008(-0.033,0.016)	0.522(0.221,0.824)
	logSRL	178	0.038	0.002	-0.094(-0.155,-0.034)	2.690(1.951,3.428)
MTCM	logSLA	238	0.006	0.227	0.001(-0.001,0.003)	1.194(1.154,1.234)
	logLT	238	0.063	0.000	0.017(0.009,0.026)	0.015(-0.152,0.182)
	logLDMC	238	0.014	0.064	-0.016(-0.033,0.001)	1.577(1.241,1.913)
	logLNC	139	0.053	0.000	-0.005(-0.008,-0.002)	1.206(1.153,1.258)
	logLPC	139	0.017	0.045	0.010(0.000,0.020)	0.427(0.230,0.623)
	logRNC	139	0.098	0.000	-0.011(-0.015,-0.006)	0.841(0.760,0.922)
	logRPC	139	0.110	0.000	0.024(0.015,0.032)	0.882(0.713,1.051)
	logSRL	178	0.011	0.112	0.018(-0.004,0.041)	1.903(1.459,2.346)
AP	logSLA	238	0.015	0.061	0.000(0.000,0.000)	1.144(1.118,1.171)
	logLT	238	0.099	0.000	0.000(0.000,0.001)	-0.606(-0.716,-0.495)
	logLDMC	238	0.024	0.002	0.000(-0.001,0.000)	2.177(1.915,2.440)
	logLNC	139	0.092	0.000	0.000(0.000,0.000)	1.386(1.352,1.421)
	logLPC	139	0.049	0.001	0.000(0.000,0.001)	-0.001(-0.132,0.130
	logRNC	139	0.099	0.000	0.000(0.000,0.000)	1.187(1.133,1.242)
	logRPC	139	0.116	0.000	0.000(0.000,0.001)	0.102(-0.012,0.216)
	logSRL	178	0.057	0.000	0.001(0.000,0.001)	0.993(0.700,1.286)
GSP	logSLA	238	0.014	0.072	0.000(0.000,0.000)	1.144(1.116,1.173)
	logLT	238	0.093	0.000	0.000(0.000,0.001)	-0.612(-0.728,-0.495)
	logLDMC	238	0.023	0.023	0.000(-0.001,0.000)	2.184(1.909,2.459)
	logLNC	139	0.090	0.000	0.000(0.000,0.000)	1.390(1.353,1.426)
	logLPC	139	0.047	0.001	0.000(0.000,0.001)	-0.009(-0.147,0.128)
	logRNC	139	0.095	0.000	0.000(0.000,0.000)	1.192(1.134,1.249)
	logRPC	139	0.113	0.000	0.001(0.000,0.001)	0.091(-0.028,0.211)
	logSRL	178	0.057	0.000	0.001(0.000,0.001)	0.961(0.654,1.268)
PET	logSLA	238	0.052	0.000	0.000(-0.001,0.000)	1.383(1.267,1.499)
	logLT	238	0.126	0.000	-0.003(-0.004,-0.002)	1.101(0.622,1.580)
	logLDMC	238	0.124	0.000	0.005(0.003,0.007)	-0.980(-1.989,0.029)
	logLNC	139	0.060	0.000	0.001(0.000,0.001)	0.996(0.842,1.150)
	logLPC	139	0.077	0.000	-0.002(-0.003,-0.001)	1.504(0.939,2.070)
	logRNC	139	0.015	0.058	0.000(0.000,0.001)	0.806(0.555,1.057)
	logRPC	139	0.022	0.023	-0.001(-0.002,0.000)	1.029(0.504,1.553)
	logSRL	178	0.064	0.000	-0.005(-0.007,-0.003)	4.169(2.889,5.450)

Korean pine functional traits were log_10_ transformed (log). Linear regression analyses were performed using the least squares method. *X* indicates geographic and climatic factors, and *Y* indicates the logarithmic value of Korean pine traits. *n*, number of samples; *R*^*2*^, coefficient of determination; *P*, significance probability of the slope test. Underlined values indicate significant linear correlations at *P* < 0.05. Numbers inside parentheses following the calculated *Slope and Intercept* values indicate the upper and lower confidence limits (UCL and LCL, respectively) of the 95% confidence interval.

### Correlations in functional traits of Korean pine

In addition to the correlations with geographic and climatic factors, there were also correlations among Korean pine functional traits (Table 4). Results evidenced significant correlations among Korean pine traits (*P* < 0.001 or *P* < 0.05), and the mean *R*^*2*^ value (0.116) indicated a low linear explanation ratio. The highest *R*^*2*^ (0.216) was found between RNC and RPC, whereas the *R*^*2*^ between SRL and RPC was only 0.038. Slope comparisons revealed that SLA and LT had a negative linear relationship with LDMC and LNC, LT showed a negative linear relationship with LNC and RNC, LDMC showed a negative linear relationship with LPC, and negative linear correlations were also found between LNC and LPC, RNC and RPC, LNC and RPC, and LPC and RNC. The remaining Korean pine traits showed positive linear relationships among them.

**Table 4:** Linear correlation analysis of Korean pine functional traits at different latitudes.

*X*	*Y*	*n*	*R*^*2*^	*P*	*Slope*	*Intercept*
logSLA	logLT	238	0.158	0.000	1.707(1.203,2.211)	-2.320(-2.910,-1.730)
	logLDMC	238	0.103	0.000	-2.702(-3.721,-1.682)	5.053(3.860,6.246)
	logLNC	139	0.147	0.000	-0.512(-0.670,-0.355)	1.901(1.716,2.085)
	logLPC	139	0.084	0.000	1.430(0.826,2.034)	-1.446(-2.153,-0.739)
	logRPC	139	0.117	0.000	1.526(0.992,2.060)	-1.365(-1.990,-0.740)
logLT	logLDMC	238	0.097	0.000	-0.609(-0.847,-0.371)	1.695(1.610,1.781)
	logSRL	178	0.068	0.000	0.675(0.356,0.993)	1.764(1.649,1.878)
	logLNC	139	0.086	0.000	-0.091(-0.129,-0.053)	1.272(1.258,1.286)
	logLPC	139	0.071	0.000	0.306(0.165,0.448)	0.326(0.275,0.377)
	logRNC	139	0.198	0.000	-0.219(-0.276,-0.163)	0.977(0.957,0.997)
	logRPC	139	0.211	0.000	0.475(0.358,0.593)	0.574(0.531,0.616)
logLDMC	logLNC	139	0.128	0.000	0.057(0.038,0.076)	1.194(1.158,1.230)
	logLPC	139	0.084	0.000	-0.170(-0.242,-0.099)	0.549(0.412,0.687)
logSRL	logRPC	139	0.038	0.002	0.078(0.028,0.128)	0.300(0.219,0.380)
logLNC	logLPC	139	0.157	0.000	-1.464(-1.898,-1.030)	2.132(1.567,2.697)
	logRNC	139	0.177	0.000	0.669(0.485,0.853)	0.177(-0.063,0.416)
	logRPC	139	0.144	0.000	-1.264(-1.658,-0.870)	2.065(1.552,2.578)
	logSRL	178	0.038	0.000	0.485(0.172,0.797)	1.342(1.201,1.483)
logLPC	logRNC	139	0.062	0.000	-0.107(-0.160,-0.053)	1.072(1.056,1.087)
	logRPC	139	0.048	0.000	0.197(0.084,0.309)	0.375(0.343,0.408)
logRNC	logRPC	139	0.216	0.000	-0.976(-1.213,-0.738)	1.442(1.193,1.692)

Korean pine trait values were log_10_-transformed (log). Linear regression analysis was performed using the least squares method. The *X* and *Y* are Korean pine phenotypes, *n* is the number of samples, *R*^*2*^ is the coefficient of determination, and *P* is the significance probability of the correlation test. The numbers inside the parentheses following *Slope* and *Intercept* values indicate the upper and lower confidence limits (UCL and LCL, respectively) of the 95% confidence interval.

## Discussion

### Effects of geographic and climatic factor variations on Korean pine functional traits

Our results showed that not all Korean pine functional trait responses to geographic and climatic factors were significant. Among the geographic factors, the effects of latitude and altitude on Korean pine functional traits were obvious; however, longitude only correlated with LDMC and SRL, and its effects on other traits were not obvious. The reason might be that changes in temperature and precipitation were more obvious with increasing latitude and altitude than with changing longitude. Some studies have shown that changes in climatic conditions caused by changes in latitude are the main cause for changes in individual and community traits [26], which is in congruence with our results. A recent study showed that herbivory decreased with latitude and increased with temperature in the Northern Hemisphere [49], which is similar to the results obtained in our study. Some other studies suggest that both latitude and longitude cause changes in climatic conditions [27], which is not supported by our results. This might be because of the narrow longitude span of Korean pine in China, which does not allow the capturing of obvious variations in climatic factors. On the other hand, changes in the characteristics of Korean pine along its vertical distribution were obvious. It has been suggested that changes in temperature and precipitation resulting from increasing altitude were the main reason for Korean pine functional trait variations [31,38], which is consistent with the findings in the present study. Concurrently, previous studies also indicated that the main reasons for habitat heterogeneity were elevation and terrain, whereas the contribution from increasing latitude were not obvious [27,50]. The present study found that Korean pine functional traits were linearly correlated with both latitude and altitude, not ruling out the possibility of interaction between these two factors. However, further studies are required to determine which factor had a major impact.

Moisture also affects the responses of Korean pine functional traits. Northeast China is affected by maritime air masses in the summer, and thus, has abundant rainfall. In the present study, GSP significantly correlated with all Korean pine traits except SLA, suggesting that this pine species is especially affected by moisture during its growth period. The limiting effects of precipitation and temperature on plant growth are key issues discussed in numerous studies. It has been suggested that in mid-latitude regions, the main limiting factor for plant growth and community distribution is precipitation; however, in high-latitude regions, the limiting effects of temperature are obvious [23]. The results of the present study are consistent with the above findings from previous studies.

Among temperature factors, GST and MTCM had clear effects on Korean pine, whereas the effect of MAT was not obvious. Because Northeast China is dually affected by high-altitude monsoons and maritime air masses and is subject to wide temperature variations throughout the year, changes in MAT might not explain the habitat conditions of Korean pine. It has been suggested that the lowest temperature is limiting for an organism survival [46], and GST in biomass accumulation is even more significant [5]. In fact, GST and MTCM reflect changes between the highest (summer) and the lowest (winter) temperatures in a year, and the results obtained in the present study also evidenced significant relationships between Korean pine functional traits and these two factors. A balanced condition between water dissipation and heat in a region is reflected in PET, which is closely related to MAT [40,51]. In the present study, PET showed significant correlations with all Korean pine traits except RNC, indicating that the degree of water evapotranspiration and heat level not only affected ecological regulation abilities such as absorption, transport, and nutrient and energy storage but also clearly affected the morphology of tissues and organs as well as their energy consumption. Both PET and MAT have been suggested to positively correlate with the leaf life span, which mainly explained the regional differences in functional traits [52].

### Relationships among Korean pine functional traits

The correlation between Korean pine functional traits reflected its adopted ecological regulation strategy in response to changes in environmental conditions. Although SLA and LT showed a significant positive linear correlation, both showed a significant negative linear correlation with LDMC: as SLA decreased with increasing latitude, LT decreased and LDMC increased. These results indicated that Korean pine decreased leaf area and thickness while increasing leaf dry matter content to resist the negative impacts caused by increasing latitude and decreasing temperature. Some studies suggested a close relationship between SLA, LT, and LDMC [53,54]. The normal metabolic capacity is maintained by decreasing water loss during respiration and increasing leaf tissue density [14]. Although SLA significantly correlated with altitude, GST, and PET, no obvious correlation was found between SLA and longitude, latitude, and other temperature and precipitation factors, suggesting that the physiological maintenance mechanism adopted by Korean pine mainly responded to latitude changes. However, these changes aimed to cope with the changes in growth season temperature and potential evapotranspiration caused by regional altitude differences. Studies have used SLA to determine plant photosynthetic capacity and the potential growth rate [9,16], and temperature had an obvious limiting effect on plant photosynthesis and respiration [22]. In the present study, SLA responses to GST reflected the environmental adaptability of Korean pine in maintaining normal photosynthesis and respiration capacities during growth. Some studies have considered LDMC as the leaf construction cost and the ability of leaves to store energy and nutrients [52] and LT as the light acquisition ability of plants and the basis for determining the CO_2_ diffusion pathway and resource utilization strategies [54]. Other studies used changes in LDMC and SLA as the basis for determining the ability of plants to absorb soil elements [55]. In the present study, Korean pine grew in the mid-latitude region, and SLA and LT decreased with decreasing temperature; therefore, photosynthetic and respiratory capacities decreased. It has been suggested that under such conditions, leaf life span increases, which is an adaption strategy of evergreen and coniferous species in response to adverse environments [1]. In addition, another study indicated that the interspecific level herbivory had a hump-shaped relationship with leaf lifespan and a positive relationship with leaf size, and nutritional traits have little relationship to herbivory [56]. This indicates that the relationship between functional traits is interrelated between species.

The trends of changes in Korean pine leaf nitrogen and phosphorus elements were similar to those of roots, suggesting that their responses to latitude variation were similar, and there was a significant correlation between them. Nitrogen is a necessary material for the synthesis of photosynthetic elements, and both nitrogen and phosphorus provide energy and material for enzymatic photophosphorylation reactions [57]. In the present study, LNC showed a significant positive linear correlation with RNC, which explained root absorption, nitrogen transport, and leaf photosynthesis to provide raw materials; on the other hand, root growth and development requires material and energy, and thus roots have high nitrogen content. However, LNC showed a negative linear relationship with LPC and RPC, indicating that Korean pine reduced leaf and root energy consumption during respiration by decreasing phosphorus content with increasing latitude. In addition, LNC showed a negative linear relationship with SLA and LT, suggesting a reduced leaf area, which is considered a trait for species with a long leaf life span and low photosynthetic capacity [52]. It has been suggested that leaf nitrogen and phosphorus contents indicate soil nutrient limitation. The results of the present study suggest that phosphorus limitation is more pronounced in this area, and an increase in latitude did not result in decreased leaf and root nitrogen contents. This might be related the abundant rainfall and low mean temperature of Northeast China, which leads to nitrogen limitation and to a slow-growth strategy. Nitrogen, photosynthetic and respiratory capacities, and leaf morphology are related, and therefore, studies have linked plant’s nitrogen-use-strategy with herbivore grazing to explain material circulation and energy loss in the food web [9].

Analyzing the relationship between functional traits and geographical and climatic factors of Korean pine also yielded reference values for the regeneration and protection of Korean pine species in northeast China. Unlike previous studies that have focused on individual growth and biomass accumulation, analyzing the functional traits in the present study allows emphasizing the limiting effect of external environmental conditions, such as different geographical factors, temperature, and precipitation, on the growth of Korean pine. Our results could help select the appropriate precipitation and altitude areas to perform manual regeneration, planting, and management of Korean pine, which can enhance the efficiency of the protection of Korean pine forest.

## Conclusions

This study analyzed Korean pine functional trait responses to latitudinal variation in Northeast China and concluded that differences in Korean pine functional trait responses to latitude were mainly because of the effects of climatic factors resulting from geographic variations. Korean pine functional trait responses to AP, GSP, and PET were the most obvious, followed by responses to altitude and latitude, and finally to GST and MTCM. Differences in Korean pine functional traits at different latitudes were noteworthy and correlations between traits were significant, which reflect the ecological adjustment measures by Korean pine to adapt to changes in environmental conditions. Geographic and climatic factors clearly contributed to regional differences in Korean pine functional traits. Precipitation, temperature, evapotranspiration, and element contents were the main factors limiting Korean pine functional traits.

We studied the functional traits of Korean pine species, which, as mentioned above, is determined by the status of Korean pine in the forest community in Northeast China. Although the experimental results have certain value for the protection of Korean pine, the limitations are still evident in the validation of a functional trait theory. For example, the functional traits of a single species are not exactly the same as those of a community or ecosystem, and this problem will be analyzed in future studies.

## Supporting information

S1 File File containing raw data of study subjects (Supporting Information2017-07-26.xlsx). (XLSX)
